# Metabolic Imaging Biomarkers of Response to Signaling Inhibition Therapy in Melanoma

**DOI:** 10.3390/cancers16020365

**Published:** 2024-01-15

**Authors:** Pradeep Kumar Gupta, Stepan Orlovskiy, Fernando Arias-Mendoza, David S. Nelson, Aria Osborne, Stephen Pickup, Jerry D. Glickson, Kavindra Nath

**Affiliations:** 1 Molecular Imaging Laboratory, Department of Radiology, University of Pennsylvania, Philadelphia, PA 19104, USA; pradeep.gupta@pennmedicine.upenn.edu (P.K.G.); stepan.orlovskiy@pennmedicine.upenn.edu (S.O.); fernando.arias-mendoza@pennmedicine.upenn.edu (F.A.-M.); dsnelson@pennmedicine.upenn.edu (D.S.N.); ariaosb@sas.upenn.edu (A.O.); pickup@pennmedicine.upenn.edu (S.P.); glickson@pennmedicine.upenn.edu (J.D.G.); 2 Advanced Imaging Research, Inc., Cleveland, OH 44114, USA

**Keywords:** BRAF, dabrafenib, melanoma, magnetic resonance spectroscopy, cancer biomarkers, response prediction

## Abstract

**Simple Summary:**

In vivo, ^1^H and ^31^P magnetic resonance spectroscopy were used to study human melanoma models to monitor the metabolic effects of dabrafenib therapy, an inhibitor of the hyperactive BRAF protein in melanoma. Four human melanoma cell lines with diverse responses to dabrafenib were tested, from least to most responsive: WM3918 < WM9838BR < WM983B < DB-1. Differences in the melanoma models’ tumor concentrations of lactate and alanine and the bioenergetics-related metabolites nucleoside triphosphates (NTP) and inorganic phosphate correlated with their diverse therapeutic responses to dabrafenib. Our results demonstrated that dabrafenib induced metabolite changes that occur soon after treatment initiation preceding response (e.g., tumor volume reduction); thus, these metabolites can be considered biomarkers of therapeutic response to dabrafenib in melanoma.

**Abstract:**

Dabrafenib therapy for metastatic melanoma focuses on blocking growth-promoting signals produced by a hyperactive BRAF protein. We report the metabolic differences of four human melanoma cell lines with diverse responses to dabrafenib therapy (30 mg/kg; oral): WM3918 < WM9838BR < WM983B < DB-1. Our goal was to determine if metabolic changes produced by the altered signaling pathway due to *BRAF* mutations differ in the melanoma models and whether these differences correlate with response to treatment. We assessed metabolic changes in isolated cells using high-resolution proton magnetic resonance spectroscopy (^1^H MRS) and supplementary biochemical assays. We also noninvasively studied mouse xenografts using proton and phosphorus (^1^H/^31^P) MRS. We found consistent changes in lactate and alanine, either in isolated cells or mouse xenografts, correlating with their relative dabrafenib responsiveness. In xenografts, we also observed that a more significant response to dabrafenib correlated with higher bioenergetics (i.e., increased βNTP/Pi). Notably, our noninvasive assessment of the metabolic status of the human melanoma xenografts by ^1^H/^31^P MRS demonstrated early metabolite changes preceding therapy response (i.e., tumor shrinkage). Therefore, this noninvasive methodology could be translated to assess in vivo predictive metabolic biomarkers of response in melanoma patients under dabrafenib and probably other signaling inhibition therapies.

## 1. Introduction

Melanoma, the most aggressive and life-threatening skin cancer, is the fastest-increasing form of human cancer in the United States and among Caucasian populations throughout the world [1]. Melanoma is highly curable when it stays in the primary site; however, metastatic melanoma has a poor prognosis with a median survival of six months [2]. Additionally, patients with metastatic melanoma have varying response rates to current systemic therapies, and most of these patients rapidly develop tumor resistance [2,3,4,5]. Five-year survival for patients with metastatic melanoma is less than 15%.

Although surgical treatment remains the gold standard for melanoma [6] recent advances in immunotherapy and targeted molecular therapy for metastatic melanoma show great promise [6]. Most melanoma cells are radio- and chemo-resistant, mainly due to their melanin production [7]. Therefore, melanoma treatment could include surgery, chemotherapy, immunotherapy, and radiotherapy [8,9,10]. Recently, a targeted therapy approach for metastatic melanoma focused on the mitogen-activated protein kinases (MAPK) pathway. The MAPK pathway relies upon extracellular signals (i.e., mitogens, osmotic stress, heat shock, and proinflammatory cytokines) to regulate intracellular processes like proliferation, gene expression, differentiation, mitosis, metabolism, cell survival, and apoptosis.

This targeted therapy approach resulted in the discovery of significant driver mutations and genetic subtypes [8]. It also provided the rationale for innovative therapeutic strategies targeting specific molecular mechanisms of oncogenesis in the treatment of melanoma. For example, BRAF mutant cutaneous melanoma is the most common subtype, with the V600E mutation occurring in most patients [11]. Furthermore, ample evidence currently exists on the critical role of abnormal DNA methylation in the development and progression of malignant melanoma [12,13]. DNA methyltransferases are significantly upregulated during melanoma progression [14]. More than 70 genes are hypermethylated in melanoma, including members of the MAPK pathway. Hotspot mutations of the oncogenes, BRAF, and RAS lead to constitutive signaling of the MAPK regulatory pathways, enhancing tumor growth and promoting disease progression [15,16].

BRAF and RAS are the most common alterations in cutaneous melanoma, and specific inhibitors have shown significant survival benefits [17]. BRAF and RAS have been targeted in 25% and 60% of the cases, respectively [17,18]. Vemurafenib and dabrafenib are BRAF inhibitors approved by the US Food and Drug Administration for treating metastatic melanoma, especially for patients harboring distant metastases with BRAF mutations [6]. BRAF inhibitors significantly improve both progression-free survival and overall survival, although resistance with relapse develops rapidly, often within 6 to 10 months of treatment [19].

Regarding metabolism, melanoma usually demonstrates a glycolytic phenotype (i.e., Warburg effect) [20,21,22]. The *BRAF* gene mutation produces this phenotype by rendering its product constitutively active [23,24] and triggering the MAPK pathway [25]. The enhanced activity of the MAPK pathway promotes the hypoxia-induced factor 1α (HIF-1α), a master regulator of glycolysis, thus resulting in an increased glycolytic activity [25]. In addition, BRAF also inhibits oxidative phosphorylation (OXPHOS) [26,27,28]. However, glycolysis inhibition in melanoma produces an initial adenosine triphosphate (ATP) drop, causing a reversion to OXPHOS for energy production. Furthermore, despite the documented OXPHOS inhibition by BRAF, many melanoma cells present a higher OXPHOS phenotype [28]. Although it would be simpler to categorize melanoma into glycolytic or OXPHOS phenotypes, an increasing body of evidence suggests that the nature of metabolic phenotypes in melanoma is dynamic—i.e., “metabolic plasticity” [29,30,31].

This report focuses on the response of human melanoma cancer cells to the signaling inhibitor dabrafenib, which targets the mutated BRAF kinase, a crucial component of the MAPK signaling pathway [3,4,32,33]. We studied three human melanoma tumor models that express the *BRAF*-V600E mutation: WM983BR, WM983B, and DB-1. This mutation replaces the amino acid valine with glutamic acid at amino acid number 600 within the protein. The *BRAF*-V600E mutation makes up to 90% of all *BRAF* mutations, is expressed in 40–60% of all human melanoma patients, and is more common in younger patients [34]. For comparison, we also studied the WM3918 human melanoma cell line that expresses wild-type *BRAF*, *c-KIT*, *RAS*, and *CDK4* genes [35]. We aimed to determine dabrafenib’s metabolic effect in these preclinical human melanoma models and correlate this effect with the elicited tumor response [22]. To do this, we studied isolated cells from the melanoma cell lines and created mouse xenografts by inoculating the cells in athymic nude mice. After treating the melanoma models with dabrafenib, we used ^1^H and ^31^P magnetic resonance spectroscopy (MRS) and other methods to detect and quantify their metabolic response to dabrafenib.

## 2. Materials and Methods

### 2.1. Reagents

MEM-α, glutamine, HEPES, penicillin-streptomycin, trypsin-EDTA, DPBS, sodium pyruvate, and HBSS were purchased from Thermo Fisher Scientific (Waltham, MA, USA; Invitrogen brand). FBS was purchased from HyClone Laboratories, Inc. (Logan, UT, USA). Dabrafenib was purchased from LC Laboratories (Woburn, MA, USA). 3-aminopropylphosphonate (3-APP) was purchased from AmBeed, Inc. (Arlington Heights, IL, USA). Trimethylsilyl propanoic acid (TSP) was purchased from Sigma-Aldrich, Inc. (St. Louis, MO, USA).

### 2.2. Melanoma Cell Lines

WM3918 (Accession No: CVCL_C279) and WM983BR (Accession No: CVCL_AP81) human melanoma cells were grown in a solution of MCDB153 and L-15 media (4:1 ratio) supplemented with 100 Units/mL penicillin, 100 µg/mL streptomycin, 2 mM calcium chloride, and 2% FBS. Additionally, WM983BR media was supplemented with 100 nM dabrafenib in 0.1% DMSO to maintain the resistance of the cell line. WM983B (Accession No: CVCL_6809) and DB-1 (Accession No: CVCL_D902) human melanoma cells were grown in MEM-α media supplemented with 25 mM glucose, 2 mM glutamine, 10 mM HEPES, 100 Units/mL penicillin, 0.1 mM MEM NEAA, 100 µg/mL streptomycin, and 10% FBS.

### 2.3. Cell Cultures

Isolated cells of the four human melanoma cell lines were seeded in T-75 tissue culture flasks. The flasks were incubated with 5% CO_2_ at 37 °C to allow the cells to adhere for 24 h. Vehicle (0.1% DMSO) or dabrafenib (25 nmol/L in 0.1% DMSO) were added to create control and treated flasks. After 48 h of incubation with vehicle or dabrafenib, media samples were collected from the flasks to measure extracellular glucose and lactate (below). In addition, 20 × 10^3^ cells were harvested and seeded into microplates to determine the oxygen consumption rate (OCR) and extracellular acidification rate (ECAR) as per below.

Furthermore, 15 × 10^6^ cells were harvested from control and treated flasks for the in vitro high-resolution ^1^H MRS studies below. These cells were centrifuged for 10 min at 1000× *g* rpm at 4 °C, the supernatant was discarded, and the cell pellets were washed twice with 1× phosphate-buffered saline (PBS) and stored in a −80 °C freezer. The frozen cell pellets were resuspended in 1 mL of an 80% methanol–water solution precooled to −80 °C. The suspension was then homogenized for 30 s and sonicated for 20 s. The sonicated suspension was centrifuged at 16 × 10^3^× *g* for 10 min at 4 °C. The supernatant was transferred to labeled Eppendorf tubes and freeze-dried using the Labconco FreeZone^4.5^ lyophilizer (Labconco Co., Kansas City, MO, USA). Lyophilized extracts were resuspended in 600 µL of deuterium oxide (D_2_O) containing 0.2 mmol/L TSP and transferred to a 5 mm NMR tube.

### 2.4. Extracellular Glucose and Lactate Measurements

Extracellular glucose and lactate measurements were performed using a YSI 2300 STAT PLUS Biochemistry Analyzer (YSI, Inc., Yellow Springs, OH, USA). The YSI 2365 D-Glucose and YSI 2329 L-Lactate enzyme membranes suspended in YSI 2392 NaCl solution (30 mL) were installed per instructions. Buffer solutions were prepared using the YSI 2357 Buffer Concentrate packages, and the YSI 2747 Glucose/Lactate Standard (1.8 g/L glucose, 0.45 g/L lactate) was used to calibrate the measurements.

### 2.5. Oxygen Consumption and Extracellular Acidification Rates

OCR and ECAR tests were performed using a Seahorse XFe96 Extracellular Flux Analyzer (Agilent Technologies; Santa Clara, CA, USA). The reagents for these tests were prepared following the Agilent Mito Stress Test Guide. Stock solutions (100 µM oligomycin, 100 µM FCCP, and 50 µM of a rotenone/antimycin-A) were prepared on the day of each assay using the lyophilized powders provided in the test kits. Working solutions were then prepared with the following concentrations: 15 µM oligomycin, 5 µM FCCP, and 5 µM rotenone/antimycin-A. Microplates with each melanoma cell line (20 × 10^3^ cells) were prepared, and the stepwise effect of each reagent on OCR and ECAR was recorded. The final concentrations in the microplate were 1.5 µM oligomycin, 0.5 µM FCCP, and 0.5 µM rotenone/antimycin-A. First, OCR and ECAR basal values were recorded. Next, oligomycin, an ATP synthase inhibitor, was added to determine ATP-linked respiration. Then, FCCP, an uncoupling agent, was used to maximize respiration and determine the spare respiratory capacity of the tested cell line. Finally, rotenone/antimycin-A and complex I and III inhibitors were added to terminate respiration.

The OCR and ECAR data were normalized after each assay using the viable cell numbers provided by the BioTek Cytation 5 Cell Imaging Multimode Reader (Agilent Technologies). Thirty microliters of a 20 mM Hoechst 33342 solution (Thermo Scientific, Waltham, MA, USA) were added to the rotenone/antimycin-A working solution, adjusting its volume to account for the addition of the fluorescent stain. At the end of the OCR/ECAR assay, the cell microplate was moved to the Cytation 5 instrument, where a fluorescence scan determined the viable cell number in each well of the microplate.

### 2.6. In Vitro Measurement of Intracellular Metabolites by High-Resolution ^1^H MRS

High-resolution ^1^H MRS was acquired with a PRESAT pulse sequence (water suppression with pre-saturation pulses) on a 9.4T/8.9 cm vertical bore Varian NMR spectrometer, flip angle = 45°, repetition time (TR) = 8.8 s, sweep width (SW) = 6756.8 Hz, number of point (NP) = 16,384, and averages (NT) = 128. The MestReC 6.1 post-processing software (Informer Technologies, Inc., Los Angeles, CA, USA) was used to process all MRS data. After Fourier transformation, a 1 Hz exponential filter was used to improve the apparent signal-to-noise ratio of the ^1^H MRS data and calculate peak areas with normalization by the number of protons.

### 2.7. Mouse Xenografts for the Noninvasive ^1^H and ^31^P MRS Studies

Mouse studies were performed under an approved protocol and following the guidelines of the University of Pennsylvania Institutional Animal Care and Use Committee (IACUC).

Male athymic nude mice, 4–6 weeks old, were purchased from Charles River Laboratories (Wilmington, MA, USA). Before inoculation, WM3918, WM983BR, WM983B, and DB-1 human melanoma cells were grown in a monolayer culture at 37 °C in 5% CO_2_ in MEM-α medium. Subsequently, melanoma cells were resuspended in Hank’s Balanced Salt Solution (HBSS) at 10^7^ cells/mL. Mouse xenografts were prepared via subcutaneous injection of 0.1 mL of the cell suspension into the right thigh. The human melanoma mouse xenografts were allowed to grow until the tumor reached an approximate volume of 250 mm^3,^ and the tumor assumed a hemispherical shape before further study.

After the tumor reached the optimal size, mice were randomly assigned to the control or dabrafenib arms. The control mice received vehicle alone while the dabrafenib mice received dabrafenib dissolved in vehicle (0.5% hydroxypropyl methylcellulose and 0.2% Tween 80 buffer solution in Milli-Q filtered water). Vehicle or dabrafenib (30 mg/kg) was administered by a single oral gavage daily until day five.

Forty-eight tumor-bearing male athymic nude mice were included in this study as follows: 10 WM3918 xenografts (control, *n* = 5; dabrafenib, *n* = 5), 10 WM983BR xenografts (control, *n* = 5; dabrafenib, *n* = 5), 14 WM983B xenografts (control, *n* = 6; dabrafenib, *n* = 8) and 14 DB-1 xenografts (control, *n* = 6; dabrafenib, *n* = 8).

### 2.8. In Vivo ^1^H and ^31^P MRS Experiments

MRS data were acquired on days 0, 2, and 5 after the start of dabrafenib treatment using a 9.4T Bruker horizontal bore spectrometer. On the day of the MRS scan, the tumor-bearing mouse was anesthetized with 1% isoflurane in 1 L/min oxygen. Breathing was monitored with a respiration pillow, and the temperature was monitored with a rectal thermometer while the animal was in the study. The core temperature of the mouse was maintained at 37 ± 1 °C by blowing warmed air into the magnet’s bore with the heating controlled by a thermal regulation system.

For the ^1^H MRS study, the animal’s tumor was positioned in a homemade ^1^H single-frequency slotted-tube resonator (15 mm outer diameter, 13 mm inner diameter, 16.5 mm depth). ^1^H MRS data were acquired with a slice-selective, double-frequency, Hadamard-selective, multiple quantum coherence (HDMD-SelMQC) transfer pulse sequence to edit lactate and alanine by canceling the rest of the ^1^H signals, especially those from overlapping lipid signals [36]. The following acquisition parameters were used: TR = 4 s, NP = 1000, NT = 32, SW = 4000 Hz, and flip angle = 90°. A localized water signal was also acquired using a similar slice without water suppression (TR = 4 s, NT = 4) to normalize the lactate and alanine signal to the water content in the same tumor volume.

^31^P MRS data were acquired as previously described [37]. Immediately before the ^31^P MRS scan, the mouse was injected intraperitoneally with 400 µL of a solution of 3-APP (300 mg/mL in water). Subsequently, the mouse’s tumor was positioned in a homemade dual-frequency (^1^H/^31^P) slotted-tube resonator (10 mm diameter). ^31^P MRS data were acquired using the Image Selected In vivo Spectroscopy (ISIS) pulse sequence with the following parameters: TR = 4 s, NT = 32; SW = 7979 Hz; offset frequency = 430, NP = 512; and flip angle = 90°. ^1^H-decoupled ^31^P MRS spectra were acquired using ^1^H-irradiation with power = 3.12 W and duration = 64.16 ms. A scout image was initially acquired to minimize contamination of tumor metabolite signals with exogenous signals from muscle.

NUTS 1D v. 20070706 (Anasazi Instruments, New Palestine, IL, USA) and MestReC 6.1 were used to process the in vivo MRS data. A 10 Hz and 40 Hz exponential filter were used to improve the apparent signal-to-noise ratio of the in vivo ^1^H and ^31^P MRS data, respectively, and baseline correction was applied before plotting and calculating peak areas. Our editing ^1^H pulse sequence has been set to measure lactate and alanine optimally. In comparison, the ^31^P spectra show several ^31^P-containing signals. However, for this study, we only integrated the β signal of nucleoside triphosphates (βNTP) and inorganic phosphate (Pi) and determined the chemical shift of Pi and 3-APP. With the first two, we obtained the βNTP/Pi ratio as a parameter of bioenergetics. We use βNTP because it is the cleaner signal from this metabolite, considering that the main contributor for this signal is ATP and that the rest of the NTPs are in fast exchange with ATP. Furthermore, as described previously [37,38], we obtained intracellular and extracellular pH with the chemical shifts of Pi and 3-APP, respectively.

### 2.9. Tumor Volume Measurement

Tumor dimensions of mouse xenograft models were measured using calipers. Tumor dimensions were measured in three orthogonal directions, and the tumor volume was calculated using the equation V = π (l × w × d)/6, where l = length, w = width, and d = depth of the tumor.

### 2.10. Statistical Analysis

Data are described graphically using summary statistics (means, standard deviation [S.D.], and standard error of the mean [S.E.M.]). An independent paired/sample *t*-test was performed for statistical analysis using SPSS 20 (IBM Corp., Armonk, NY, USA). A *p*-value ≤ 0.05 was considered significant. Individual tumor volume was normalized by the mean volume of Day 0 in each group. These mean values were then averaged across tumors to obtain mean values for individual days. The S.E.M. and the *p*-values of the paired *t*-test were reported on each group for pairwise comparison between days.

## 3. Results

**Figure 1:**
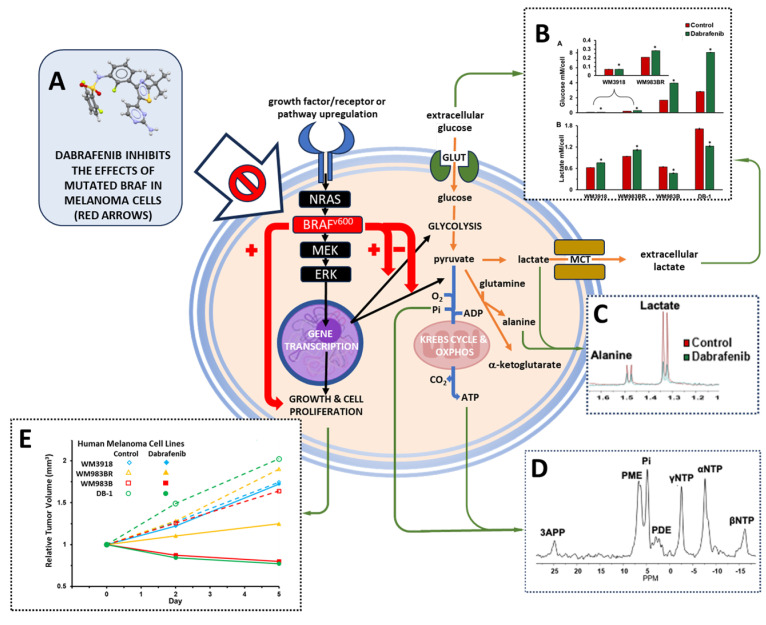
Schematic representation of the melanoma research. The mutated BRAF protein (BRAF^V600E^; red rectangle) has positive effects on growth and cell proliferation as well as on glycolysis (red arrows with positive sign) while its effect on the complete pyruvate oxidation by the mitochondria is negative (red arrow with negative sign). In contrast, dabrafenib (**A**; ball-and-stick model from [40]) inhibits the effects of the mutated BRAF protein, reducing growth and cell proliferation while controlling the Warburg effect (large white block arrow with the prohibition sign). The orange arrows highlight the enhanced metabolic effects of BRAF^V600E^, while blue arrows represent its inhibitory metabolic effects. The green arrows underscore representative examples of our results measuring extracellular glucose and lactate values (**B**; see Figure 4 for full explanation of the panel), intracellular lactate and alanine values using noninvasive ^1^H MRS (**C**; see Figures 2 and 5), bioenergetic-related metabolites also measured noninvasively by ^31^P MRS (**D**; see Figure 6), and tumor growth (**E**; see Figure 7 and Table 2), In this study, we assessed the values of these parameters in four models of human melanoma with diverse responses to dabrafenib (WM3918 < WM9838BR < WM983B < DB-1). We aimed to determine the metabolic effects of dabrafenib in melanoma and their correlation with response.

Figure 1 emphasizes the effects of the mutated BRAF protein (BRAF^V600E^) on growth, cell proliferation, and glycolysis (positive), and on the complete pyruvate oxidation by the mitochondria (negative). These metabolic outcomes are related to the Warburg effect [39]. As in most cancers, melanoma has an increased glucose uptake by its transporter (GLUT), which is metabolized through glycolysis to obtain pyruvate. However, most of the carbon backbone of pyruvate does not enter the mitochondria for further oxidation. Instead, it is reduced to lactate and concomitantly extruded to the extracellular space using the monocarboxylate transporter (MCT). Additionally, alanine can be produced from the surplus of pyruvate using amino groups from other amino acids, mainly glutamine. 

### 3.1. Impact of Dabrafenib on Intracellular Lactate and Alanine in Human Melanoma Cell Lines

**Figure 2:**
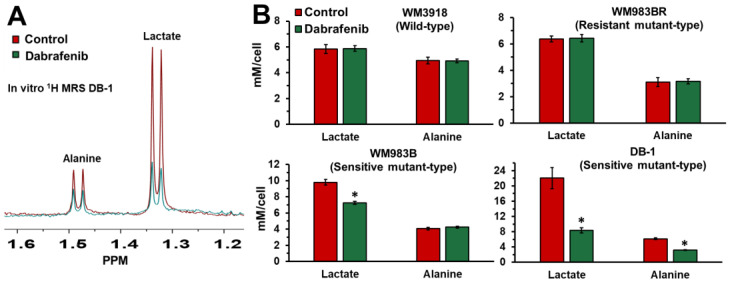
(**A**) Representative high-resolution ^1^H MRS spectra of alanine and lactate in the DB-1 cell line. The spectrum of control cells is in red, and the spectrum of dabrafenib-treated cells is in green. (**B**) Intracellular lactate and alanine concentrations in mM/cell measured by ^1^H MRS in isolated human melanoma cells. An equal number of cells (1.5 × 10^7^ cells) was used for each analysis. The asterisk (*) represents a statistically significant difference (σ = 0.05) between control (*n* = 3) and treated (*n* = 3) groups.

To determine the effect of BRAF inhibition on the metabolism of human melanoma cells, we performed in vitro ^1^H MRS at 9.4T on four melanoma cell lines treated with dabrafenib, a wild-type (WM3918), a resistance mutant-type (WM983BR), and two sensitive mutant-types (WM983B and DB-1). Dabrafenib may modify a large spectrum of metabolic pathways in these cells. However, we focused on lactate, alanine, bioenergetics, and pH, which are related to glycolysis and the Warburg effect. As shown in Figure 2, lactate and alanine varied depending on the cell line’s sensitivity to dabrafenib. Our findings indicate that treatment with 25 nM dabrafenib for 48 h produced no significant difference in the intracellular lactate and alanine concentrations in the resistant wild-type WM3918 and resistant mutant-type WM983BR human melanoma cells. However, in the sensitivity mutant-type WM983B human melanoma cells, there was a significant decrease in the synthesis of lactate, while in the sensitivity mutant-type DB-1, there was a significant decrease in the synthesis of lactate and alanine with dabrafenib.

### 3.2. Impact of Dabrafenib on Oxygen Consumption Rate (OCR) and Extracellular Acidification Rate (ECAR) in Melanoma Cell Lines

Table 1 summarizes the statistical analysis of the basal and stressed OCR and ECAR results and shows their changes in untreated vs. dabrafenib-treated melanoma cell lines. The results presented in Table 1 demonstrate that the wild-type WM3918 cells and resistant melanoma cell lines WM983BR maintain their untreated glycolytic and oxidative rates and overall energy in the presence of dabrafenib. However, WM3918 differs from WM983BR as the former is more glycolytic and less quiescent. In contrast, the sensitive WM983B cells shift from an aerobic to a low-energetic, glycolytic phenotype when treated with dabrafenib, while the DB-1 cells change from the highest energetic state of all cell lines to a less energetic, more quiescent phenotype in the presence of dabrafenib.

**Figure 3:**
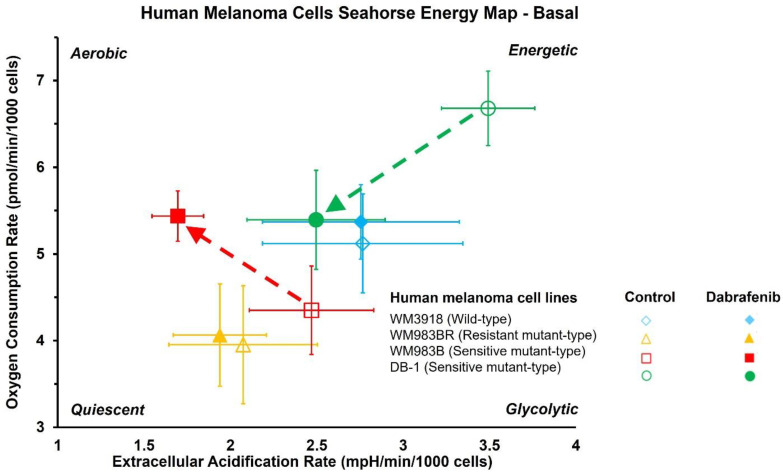
Dabrafenib’s effect on Oxygen Consumption Rate (OCR) vs. Extracellular Acidification Rate (ECAR) in melanoma cell lines under basal conditions. Untreated (open symbols) and dabrafenib-treated cells (solid symbols; *n* = 24 per group) are shown. The dashed lines indicate significant shifts in the cell energy phenotype between control and dabrafenib treatment (see Table 1).

Figure 3 depicts the OCR vs. ECAR results in the four human melanoma cell lines during basal conditions in untreated (empty symbols) and dabrafenib-treated cells (solid symbols). The Figure shows that the dabrafenib-treated resistant WM3918 (blue) and WM983BR human melanoma cell lines (yellow) demonstrated little or no difference in their basal OCR and ECAR results compared to untreated cells. However, the dabrafenib-treated sensitive WM983B human melanoma cells (red) exhibited significantly higher basal OCR with a lower basal ECAR than the untreated cells. Furthermore, the dabrafenib-treated DB-1 cells (green), also sensitive to dabrafenib, exhibited significantly lower basal and stressed OCR and ECAR than the control cells.

### 3.3. Dabrafenib Impact on Extracellular Glucose Consumption and Lactate Flux in Melanoma

**Figure 4:**
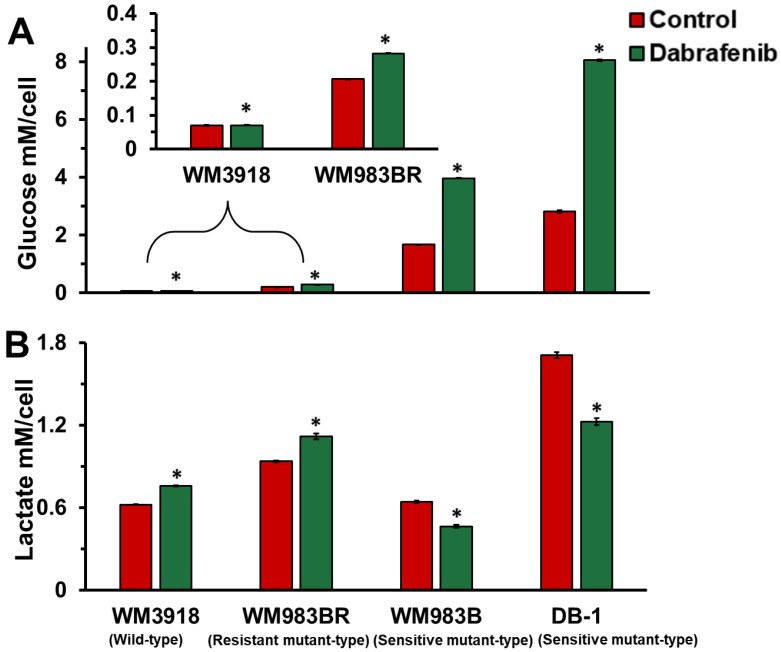
Measurement of the extracellular flux of (**A**) glucose and (**B**) lactate in wild-type (WM3918), resistant mutant-type (WM983BR), and sensitive mutant-type (WM983B, and DB-1) melanoma cells incubated in the untreated (control; red columns) or treated (dabrafenib [25 nmol/L]; green columns) at 48 h. The inset in (**A**) expands 20 times the data for WM3918 and WM983BR. Error bars represent the SEM. An asterisk (*) represents a statistically significant difference (σ = 0.05) between the control (*n* = 3) and dabrafenib-treated (*n* = 3) groups.

The extracellular lactate flux measurement depicted in Figure 4 additionally showed differences in the response between untreated and dabrafenib-treated cells (25 nM for 48 h). While lactate flux was significantly increased in the resistant melanoma cell lines (WM3918 and WM983BR), a decrease in extracellular lactate flux was demonstrated for both sensitive human melanoma cells (WM983B and DB-1).

### 3.4. Impact of Dabrafenib on Intracellular Lactate and Alanine Values in Melanoma Xenografts

**Figure 5:**
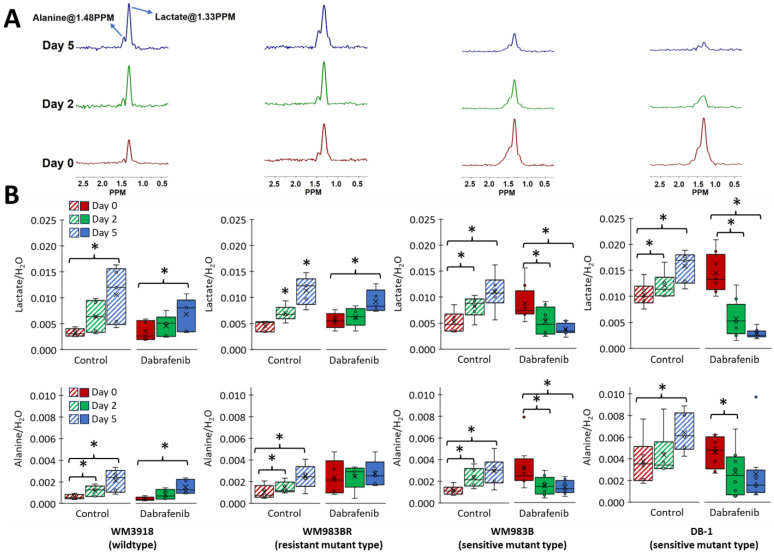
(**A**) Representative ^1^H MRS with Hadamard-selective, multiple quantum coherence (HDMD-SelMQC) transfer pulse sequence to measure lactate and alanine at Day 0 (red), Day 2 (green), and Day 5 (blue) after treatment with dabrafenib in each human melanoma xenografts model. (**B**) Box-and-whisker plots of lactate and alanine normalized by the water content in the same volume (i.e., lactate/H_2_O and alanine/H_2_O) were determined from the ^1^H MRS spectra depicted in (**A**) in control (striped bars) and dabrafenib-treated groups (solid bars). The asterisk (*) represents a statistically significant difference (σ = 0.05).

Mice with implanted melanomas on their flanks were studied noninvasively using ^1^H-MRS before and after treatment with dabrafenib. The effect of treatment with dabrafenib on the intracellular concentrations of lactate and alanine was monitored as a function of time following drug administration at days 0, 2, and 5 concerning the initial dabrafenib administration. We used the HDMD-SelMQC transfer pulse sequence, which is selective to determine the methyl resonance of lactate and alanine. Total levels of lactate and alanine were obtained by integrating the peak areas of these resonances (Figure 5). There was no effect in the intracellular lactate values of WM983BR and WM3918 human melanomas between day 0 and day 2. However, DB-1 and WM983B human melanoma xenografts treated with dabrafenib significantly decreased tumor lactate between day 0 and day 2. Furthermore, the low mean values of lactate and alanine in the two sensitive melanoma xenografts were maintained from day 2 through day 5, making them significant against day 0 but non-significant against day 2 of dabrafenib treatment (Figure 5).

### 3.5. Impact of Dabrafenib on Bioenergetics and pH in Melanoma Xenografts

**Figure 6:**
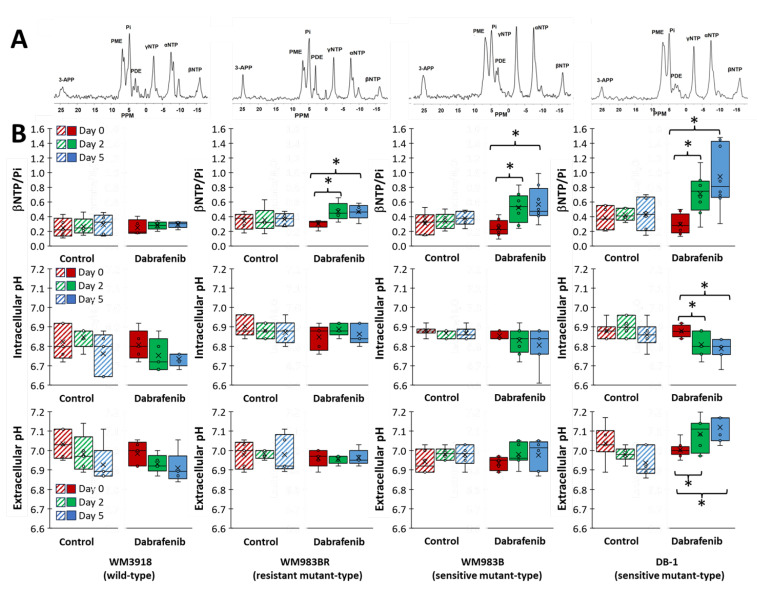
(**A**) Representative in vivo localized ^31^P MR spectra of human melanoma xenografts (cell line name at the bottom). (**B**) Bar plots of bioenergetics (βNTP/Pi), and intra- (pHi) and extracellular pH (pHe) determined from the ^31^P MRS measurements in control (striped bars) and dabrafenib treated groups (solid bars) on Day 0 (red), Day 2 (green), and Day 5 (blue). An asterisk (*) represents a statistically significant difference (σ = 0.05). Assignments in (**A**): 3-APP, 3-aminopropylphosphonate; PME, phosphomonoesters; Pi, inorganic phosphate; PDE, phosphodiesters; γ-, α-, β-NTP, the γ, α, and β-phosphates of nucleoside triphosphates.

The ^31^P spectrum of the tumor models consists of well-resolved low-field resonances, including the three phosphate moieties of NTP and Pi. Given that the NTP signals predominantly originate from ATP, that there is a rapid exchange of the terminal phosphates of the NTPs, and that the βNTP signal does not have other overlapping signals nearby, we considered that the βNTP/Pi ratio represents most accurately the in vivo bioenergetic state of the tumors. Furthermore, utilizing the ^31^P MRS chemical shift differences between Pi and αNTP and 3-APP and αNTP, we measured pHi and pHe, respectively. The externally added 3-APP has varied signal intensities because its dose was not adjusted for variations in animal weight. Moreover, since this was a bolus administration, signal intensities may have varied due to clearance rate differences in individual animals and the duration of the study. Since the critical factor for using this exogenous agent is the signal frequency to determine external pH, as far as there is a measurable signal, its intensity is less critical towards the rigor of quantification of this physiological parameter. Panel A of Figure 6 shows representative noninvasive spectra localized in the tumor of the four xenografts (assignments in the figure caption). Panel B depicts box-and-whisker plots of the values of the ratio βNTP/Pi and intracellular (pHi) and extracellular pH (pHe) assessed from the ^31^P MRS data as previously described. As shown in the Figure, only the DB-1-sensitive xenograft showed significant changes in pH under dabrafenib treatment. In this xenograft, the intracellular milieu became significantly acidic, while the extracellular milieu became significantly alkaline. Interestingly, dabrafenib increased significantly the βNTP/Pi ratio in the three mutant types regardless of whether they were resistant or sensitive to dabrafenib. These results could be considered paradoxical and should be explored further.

### 3.6. Impact of Dabrafenib on Tumor Burden in Melanoma Xenografts

**Figure 7:**
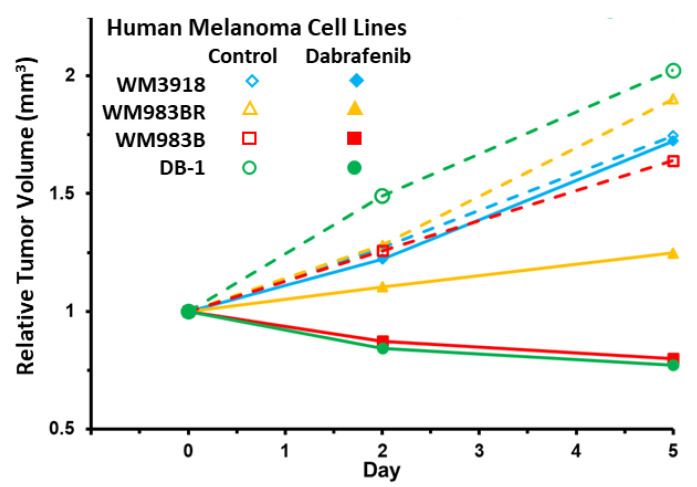
Relative tumor volume changes (in mm^3^) between Day 0, Day 2, and Day 5 of control (open symbols and dotted lines) vs. dabrafenib-treated (solid symbols and solid lines) in the human melanoma mouse xenografts.

As shown in Figure 7, control mice of all xenografts exhibited a monotonic and significant increase in tumor volume on days 2 and 5 (empty symbols and dashed lines). In contrast, the sensitive xenografts (WM983B [red] and DB-1 [green]) exhibited a progressive decrease in tumor volume following dabrafenib treatment. Furthermore, the resistant mutant-type (WM983BR [yellow]) showed a slight increase from day zero to days two and five, but these values were significantly lower compared with the same xenograft with no treatment. In comparison, the tumor volume of WM3918 wild-type (blue) had a similar behavior with and without dabrafenib. Table 2 summarizes these results and their statistical results.

## 4. Discussion

We used human melanoma cell lines with different phenotypes demonstrated by diverse degrees of dependence on the overactive BRAF protein. BRAF is an essential part of the MAPK signaling pathway, which regulates various vital cell functions, including cell growth and apoptosis (Figure 1) [41]. We found that using dabrafenib to inhibit the overactive BRAF that produces abnormal MAPK signaling profoundly modifies tumor metabolomics in melanoma phenotypes with high BRAF dependence [42]. Moreover, our results assert that the relationship between tumor metabolism and BRAF inhibition has significant translational implications. A crucial role of molecularly targeted agents inducing selective BRAF inhibition and, thus, inhibiting the abnormal MERK signaling pathway in melanoma is to restore apoptosis. Therefore, it is vital to find ways to maximize the benefits of BRAF to obtain high objective response rates early during therapy [43]. Our results show that dabrafenib treatment had little to no effect on the OCR and ECAR of the wild-type, dabrafenib-resistant WM3918 human melanoma cell line. There was also no shift in energy metabolism with treatment in WM3918 wild-type, with the cells remaining glycolytic throughout the 48 h treatment (i.e., relying on glycolysis for energy). These results were expected as this cell line does not express the V600E *BRAF* mutation that makes cancer cells susceptible to BRAF signaling inhibition.

Notably, the WM983BR human melanoma cell line showed striking metabolic and growth differences compared to the other cell lines. Although WM983BR cells express the *BRAF* mutation, these cells have acquired BRAF resistance through flexible switching among the three RAF isoforms of the ERK/MAPK signaling pathway: ARAF, BRAF, and CRAF. Through this switching, signaling progresses through MEK1/2 and ERK1/2 via kinase phosphorylation, resulting in increased gene transcription and expression of the malignant phenotype and upregulated growth of cells. This upregulated growth could be the reason why there is little to no change in OCR and ECAR in both the control and dabrafenib-treated WM983BR cells (Figure 3), with a paradoxical increase of the βNTP/Pi ratio in the dabrafenib-treated WM983BR xenografts, demonstrating an increase in bioenergetic status under dabrafenib treatment (Figure 6). Furthermore, this cell line had diminished growth compared to its untreated counterpart but still grew in the presence of dabrafenib (Figure 7). The slight but significant increase in lactate synthesis with no production of alanine in this cell line (Figure 5) coupled with an increased flux of extracellular lactate (Figure 4) suggests that this cell line could be strongly relying on glycolysis (i.e., Warburg effect) to maintain a high bioenergetic state despite of the presence of dabrafenib.

In comparison, the WM983BR cell line differs metabolically from WM983B as WM983BR maintained its bioenergetic status in the presence of dabrafenib, increased the production of alanine as well as lactate coupled to an increased extracellular lactate flux. These results suggest that WM983BR may rely on a metabolic source different from glycolysis to maintain its growth. Relative to Day 0 (Figure 5), this cell line produced the most alanine in the presence of dabrafenib compared to the remaining cell lines. This fact suggests that a possible energy source for WM983BR is glutamine, which needs to be deaminated before being a source of cellular energy and, thus, increasing alanine production (i.e., glutaminolysis).

Untreated DB-1 cells showed the highest OCR and ECAR of all the melanoma lines studied. These higher values demonstrate that DB-1 cells are the most energetic, relying heavily on OXPHOS to meet their energy requirements for growth and proliferation (Figure 3). The untreated DB-1 xenografts also showed slightly higher levels of βNTP/Pi than the rest of the xenografts supporting this concept (Figure 6). Importantly, DB-1 cells also showed the most significant decrease from energetic to quiescent in the presence of dabrafenib. DB-1 is a *BRAF* mutant, so these results demonstrate the effective dabrafenib inhibition of the BRAF kinase in this melanoma cell type.

Furthermore, these results also show that the cell line relies heavily on ERK/MAPK signaling for growth and proliferation. However, the change to a quiescent state in the in vitro studies means that the DB-1 xenografts are not utilizing the Warburg effect for cell growth and division. The quiescent state could explain, at least in part, the significant increase of the bioenergetic state demonstrated in the DB-1 xenografts (i.e., not an increase in energy production but a reduction in its utilization).

Comparatively, WM983B cells showed a shift to an anaerobic state in the presence of dabrafenib (Figure 3 and Table 1), making them more reliant on glycolysis for their energy needs. Although WM983B cells express the *BRAF* mutation, the different direction and magnitude of the shift on the energy map of OCR vs. ECAR suggests that these cells do not rely on the ERK/MAPK pathway as much as the DB-1 cells do. Previous experiments have shown a similar trend [38] in WM983B cells compared to DB-1 using similar conditions. The correlation of the WM983B energy map changes after dabrafenib treatment (Figure 3 and Table 1) with an increased βNTP/Pi (Figure 6) could also be explained by increased glycolysis improving the bioenergetic state of the cell (i.e., reducing energy utilization and increasing its production).

Our MRS results ascertain the critical role of dabrafenib in melanoma cell metabolism. We theorized that the therapy effects of drugs targeting BRAF/MERK on glycolysis and Warburg effect should be demonstrated by significantly reducing intracellular and extracellular lactate in sensitive cell lines. In contrast, the non-responding cell lines should behave as controls, maintaining or increasing the intracellular lactate content. Our results in Figure 2, Figure 4 and Figure 5 demonstrate these facts.

Alanine, the aminated product of pyruvate and a crucial product of aminotransferases, is a critical player in the glutaminolysis pathway (Figure 1). Our results in Figure 2 and Figure 5 show a decrease in alanine concentration in the presence of BRAF inhibition by in vitro and in vivo ^1^H MRS. These results suggest that BRAF inhibition reduces glutaminolysis, further altering the bioenergetic status in sensitive melanoma lines. Furthermore, a significantly increased alanine production is shown in all the untreated xenografts and is maintained in WM3918 but not in WM983BR after dabrafenib treatment. As described previously, these results suggest that glutaminolysis is a source of energy for WM3918 and could explain why this cell line does not respond to dabrafenib, but it is not the reason why WM983BR is dabrafenib insensitive.

Importantly, this information also implies that our noninvasive measurement of alanine by ^1^H MRS can serve as an in vivo biomarker of glycolysis (its carbon backbone) and glutaminolysis (its amino group) and, together with lactate, can help assess the Warburg effect, mitochondrial respiration, intracellular acid-base balance, and oxygen consumption in melanoma (Figure 2 and Figure 5). We also observed that higher total bioenergetic levels (βNTP/Pi) by ^31^P MRS correlate with increased response to dabrafenib in responsive melanoma lines (Figure 6). The more glycolytic or more energetic tumors (WM983B and DB-1) appear more responsive to dabrafenib compared to the resistant (WM983BR) and wild-type (WM3918) melanoma cell lines. The reason for this correlation between cellular energy state and the extent of glycolysis remains to be determined. However, this selective tumor de-energization could enhance tumor response to therapeutic agents such as dabrafenib.

## 5. Conclusions

In this study, we have identified metabolic biomarkers that predict early and reliably the response or the resistance to BRAF inhibition in preclinical human melanoma models. We found that inhibiting BRAF, a constitutive part of the MAPK pathway, profoundly changes tumor metabolism in melanoma phenotypes with high BRAF dependence [42]. Moreover, we verify that the relation between tumor metabolism and BRAF inhibition has the potential for significant clinical translational implications. ^1^H MRS-derived metabolic biomarkers indicate melanoma response or resistance to BRAF inhibition (Figure 2 and Figure 5). Using in vivo ^31^P MRS, we also noted that higher bioenergetic levels (i.e., high βNTP/Pi ratios) correlated with increased response to dabrafenib (Figure 6). Our findings strongly suggest that modifications of tumor metabolism are essential mechanisms responsible for the efficacy of BRAF inhibitors as therapeutic agents in melanoma. The reason for the correlation of response with cellular energy state or extent of glycolysis remains to be determined. Measurements of glycolysis and bioenergetics may serve as the basis of predictive estimates of the response of melanoma patients to an energetically demanding course of treatment. It is realistic to think that evidence about metabolism could also be valuable in monitoring and predicting responses to other cancers and treatment modalities, such as BRAF and MEK inhibitors and immunotherapy. Future studies are needed to determine the nature of the link between the observed changes and correlate with glutaminolysis, oxidative phosphorylation, and other biochemical parameters.

In summary, studying preclinical human melanoma models using in vivo and in vitro ^1^H/^31^P MRS and supporting methodology demonstrated varied metabolic differences between the models before and during dabrafenib therapy. Our study focused on therapy-related changes in glycolysis, the Warburg effect, the acid-base balance, glutaminolysis, and bioenergetics. Our results increased the understanding of the mechanistic basis of the different responses to dabrafenib depending on the melanoma phenotype. Notably, our results also demonstrated that metabolic changes during dabrafenib treatment preceded and, thus, predicted the objective demonstration of therapeutic response in the preclinical melanoma models. This treatment response prediction in preclinical models of human melanoma using noninvasive ^1^H/^31^P MRS highlights the potential clinical translation of these measurements. Clinical MR imaging systems available in most hospitals could be used to noninvasively obtain these predictive metabolic biomarkers of response in patients with metastatic melanoma treated with dabrafenib and possibly other signal inhibition therapies.

## Figures and Tables

**Table 1:** Oxygen Consumption and Extracellular Acidification Rates in Melanoma Cell Lines.

Oxygen Consumption Rate (OCR; pmol/min/cell)						
Group	Basal		*p*-Value	Stressed		*p*-Value
	Control	Dabrafenib		Control	Dabrafenib	
WM3918	5.12 ± 0.57	5.37 ± 0.43		7.47 ± 1.02	7.86 ± 1.41	
WM983BR	3.95 ± 0.68	4.06 ± 0.59		5.52 ± 0.81	5.96 ± 0.55	
WM983B	4.35 ± 0.51	5.44 ± 0.29	0.004	6.97 ± 1.13	12.6 ± 2.42	0.002
DB-1	6.68 ± 0.43	5.39 ± 0.57	0.004	15.6 ± 2.34	7.97 ± 1.64	<0.001
**Extracellular Acidification Rate (ECAR; pmol/min/cell)**						
WM3918	2.77 ± 0.58	2.76 ± 0.57		4.63 ± 0.86	4.76 ± 0.96	
WM983BR	2.07 ± 0.43	1.94 ± 0.27		3.44 ± 0.66	3.25 ± 0.52	
WM983B	2.47 ± 0.36	1.69 ± 0.15	0.002	4.73 ± 0.57	3.29 ± 0.21	0.002
DB-1	3.49 ± 0.27	2.50 ± 0.40	<0.01	5.35 ± 0.32	4.26 ± 0.46	0.009

**Table 2:** Comparison of the relative tumor volume in the control vs. dabrafenib groups of the human melanoma cell lines.

Human Melanoma Cell Lines	Relative Tumor Volume (Mean ± S.E.M.)			
		Day 0	Day 2	Day 5
WM3918	Control	1.00 ± 0.12 *	1.27 ± 0.13 **	1.75 ± 0.09 ***
	Dabrafenib	1.00 ± 0.03 *	1.22 ± 0.09 **	1.72 ± 0.17 ***
WM983BR	Control	1.00 ± 0.15 *	1.28 ± 0.18 **	1.90 ± 0.32 ***
	Dabrafenib	1.00 ± 0.08	1.10 ± 0.11	1.25 ± 0.13 ***
WM983B	Control	1.00 ± 0.13 *	1.26 ± 0.16 **	1.64 ± 0.20 ***
	Dabrafenib	1.00 ± 0.08 ^#^	0.87 ± 0.07 ^##^	0.80 ± 0.07 ^###^
DB-1	Control	1.00 ± 0.20 *	1.49 ± 0.16 **	2.02 ± 0.20 ***
	Dabrafenib	1.00 ± 0.17 ^#^	0.84 ± 0.16 ^##^	0.77 ± 0.16 ^###^

Significant difference of the mean values at * Day 0 vs. Day 2; ** Day 2 vs. Day 5; and *** Day 0 vs. Day 5 in control groups. Significant difference of the mean values at ^#^ Day 0 vs. Day 2; ^##^ Day 2 vs. Day 5; and ^###^ Day 0 vs. Day 5 in dabrafenib-treated groups.

## Data Availability

Data contained within the article are available upon request.
